# Novel genotyping algorithms for rare variants significantly improve the accuracy of Applied Biosystems™ Axiom™ array genotyping calls: Retrospective evaluation of UK Biobank array data

**DOI:** 10.1371/journal.pone.0277680

**Published:** 2022-11-17

**Authors:** Orna Mizrahi-Man, Marcos H. Woehrmann, Teresa A. Webster, Jeremy Gollub, Adrian Bivol, Sara M. Keeble, Katherine H. Aull, Anuradha Mittal, Alan H. Roter, Brant A. Wong, Jeanette P. Schmidt

**Affiliations:** Thermo Fisher Scientific, Santa Clara, CA, United States of America; University of North Carolina at Chapel Hill, UNITED STATES

## Abstract

The UK Biobank genotyped about 500k participants using Applied Biosystems Axiom microarrays. Participants were subsequently sequenced by the UK Biobank Exome Sequencing Consortium. Axiom genotyping was highly accurate in comparison to sequencing results, for almost 100,000 variants both directly genotyped on the UK Biobank Axiom array and via whole exome sequencing. However, in a study using the exome sequencing results of the first 50k individuals as reference (truth), it was observed that the positive predictive value (PPV) decreased along with the number of heterozygous array calls per variant. We developed a novel addition to the genotyping algorithm, Rare Heterozygous Adjusted (RHA), to significantly improve PPV in variants with minor allele frequency below 0.01%. The improvement in PPV was roughly equal when comparing to the exome sequencing of 50k individuals, or to the more recent ~200k individuals. Sensitivity was higher in the 200k data. The improved calling algorithm, along with enhanced quality control of array probesets, significantly improved the positive predictive value and the sensitivity of array data, making it suitable for the detection of ultra-rare variants.

## Introduction

UK Biobank is a large-scale biomedical database and research resource, containing in-depth genetic and health information from half a million UK participants [1] all genotyped on Thermo Fisher Scientific’s Applied Biosystems™ Axiom™ microarrays. About 440k individuals were genotyped on the UK Biobank Axiom array, a Research Use Only (RUO) microarray designed for the high-throughput genotyping of the UK Biobank cohort. The remaining ~50k individuals were genotyped on the UK BiLEVE Axiom microarray, a very similar, initial version of the UK Biobank array. Whole exome sequencing data for an initial subset of about 50,000 participants was made available in 2020 [2, 3]. In 2021, whole exome sequencing data for an additional 150,000 participants was released [2, 4].

In a recently published manuscript, Weedon et al. [5] compared the genotyping calls from the UK Biobank microarray to those in the initial (50k) whole exome sequencing release. Variants were binned by minor allele frequency (MAF) computed from the array calls (henceforth, cMAF). They observed that positive predictive values were at least 95% in cMAF ranges above 0.01% but dropped to 40% - 70% for cMAF ranges between 0.001% and 0.01% and dropped further to 16% for cMAF below 0.001%. This lowest cMAF group corresponded to variants with fewer than 9 individuals out of ~500,000 with non-homozygous array genotypes.

Ultra-rare variants can be difficult to genotype on microarray platforms due to the clustering algorithms used to identify the genotype [6–8]. For common variants, the location and shape of the heterozygous cluster provides powerful evidence for the accuracy of a heterozygous call. False heterozygous predictions are more likely to occur in cases where the number of true heterozygotes is too low (or zero in the most extreme case) to correctly anchor the heterozygous cluster [5, 9]. To address this problem, the Rare Heterozygous Adjusted (RHA) algorithm was introduced in 2020 as part of the standard calling algorithm for all Axiom arrays. RHA is a novel addition to the current AxiomGT1 genotyping algorithm [8], tailored to rare variants. It eliminates the majority of false heterozygous calls in rare variants while retaining >99% of true heterozygous calls.

In this study, we evaluated the effect of the RHA algorithm on the positive predictive value (PPV) of microarray genotypes in the UK Biobank cohort (the index set), using whole exome sequencing data as reference. The comparison included the subset of the UK Biobank cohort that also had whole exome sequencing data and the subset of variants included in both the index and reference sets. We compared array genotyping results before and after applying the improved algorithm, using both the initial exome data release [3] used by Weedon et al. [5] and the combined exome data for 200,000 individuals [4].

In addition to positive predictive value, we evaluated sensitivity of rare variant detection. Probesets for rare variants are particularly challenging to validate at the time of the design of an array, since the minor allele is unlikely to be found among the samples used for validation. A probeset designed from first principles may be non-responsive to the minor allele in practice, leading to low sensitivity. Knowledge of the expected minor allele frequency for rare variants has significantly improved over the last years. Therefore, we also examined whether filtering out a small set of non-responsive probesets (identified without use of the exome sequencing data) could significantly improve the sensitivity of microarray genotypes.

Together, these new methods reduced false positive heterozygous calls and increased sensitivity when genotyping rare variants.

## Methods

### Study design, participants and test methods

We performed a retrospective comparison of genotyping data of the UK Biobank cohort, comparing Applied Biosystems Axiom array genotyping calls (the index set) with the corresponding calls from whole exome sequencing (used here as the reference standard). The UK Biobank participants (55% female) were recruited from the general UK population between the years 2006 and 2010 and were 40–69 years old at the time of recruitment. The UK Biobank has received ethics approval from the North West Multi-center Research Ethics Committee (ref 11/NW/0382). At recruitment, all UK Biobank participants provided electronically signed consent. All participant records are linked-anonymized.

Genotypes from the Applied Biosystems Axiom array platform are available for the overwhelming majority (488,377) of UK Biobank participants across two arrays (UK BiLEVE for the first 49,950 participants and UK Biobank for the rest). These two arrays are very similar, sharing 95% of their content, and for both arrays, genotype calling was done in batches of about 5,000 participants [1].

The analyses in this paper center around the genotyping data for samples genotyped on the UK Biobank Axiom array. The corresponding analysis of samples genotyped on the UK BiLEVE array is presented in the supporting information.

### Processing of Axiom genotyping data

We excluded problematic variants with the same standard quality metrics used by Weedon et al. [5]: those with call rate below 95% or Hardy Weinberg P<1x10^-6^ [10]. We also excluded from analysis variants with cMAF <1% where exome sequencing and Axiom genotyping disagreed on the identity of the major allele. These few variants (<100 on either array) had extremely large discrepancies in their genotypes and calling was clearly not working correctly in at least one of the two technologies. Excluding such probesets had no effect on mean performance measures (obtained by computing performance for each variant and then calculating the mean across variants), as used by Weedon et al. However, these probesets had a large and unwarranted (since they should be filtered out of any analysis) impact on PPV when summing over all variants and samples.

The genotypes available for download from the UK Biobank site were generated prior to the release of the RHA algorithm. To obtain the identity of heterozygous calls set to “No Call” by RHA, we downloaded the sample CEL files from UK Biobank and re-ran the genotyping algorithm using the apt-genotype-axiom function from Applied Biosystems Array Power Tools (APT) v. 2.11.3 (https://www.thermofisher.com/us/en/home/life-science/microarray-analysis/microarray-analysis-partners-programs/affymetrix-developers-network/affymetrix-power-tools.html), restricting to rare variants. For direct comparison with the exome sequencing data (which is provided on human genome (hg) build 38), we converted the coordinates of the Axiom arrays from build 37 to hg38 according to the remapped location provided by dbSNP [11, 12]. Where dbSNP coordinates were not available, we used a liftover tool to calculate the position in hg38.

### Exome sequencing data

The first whole exome sequencing data release (50k) consisted of 49,936 individuals [3]. The 200k whole exome sequencing release superseded the 50k data release and provided a larger sample size. The 200k data release re-processed the sequencing data from the samples of the first release along with additional samples for a total of 200,455 samples after exclusion of withdrawn participants [4]. The 50k data were processed with a Functional Equivalence (FE) pipeline that utilized GATK [13] for both per-sample variant calling and joint-genotyping. Following Weedon et al. we used the Genomic VCF (gVCF) file for each sample (50k FE-VCF), restricted to exome sequencing depth > 15, as described by Weedon et al. [5], to reproduce their results.

The 200k data release was processed with a Functional Equivalence protocol that retained original quality scores (OQFE). Three data sources resulted:

1. Per-sample gVCF files (200k OQFE-VCF) generated using the machine-learning based variant caller DeepVariant [14];
2. A population-level VCF file (200k pVCF) generated using GLnexus [15] to joint-genotype the gVCFs;
3. 200k OQFE-PLINK, a PLINK format version of the 200k pVCF.

We used the joint-genotyped 200k OQFE-PLINK data, filtering at read depth 5. The OQFE- PLINK data is restricted to variants that are polymorphic in the whole exome sequencing data.

### Computation and summary of performance metrics

Following the example of previous work, we calculated for each variant four performance metrics: sensitivity, specificity, positive predictive value and negative predictive value. We computed these metrics for all single nucleotide variants that were both assayed on the array and had exome sequencing data. Insertions and deletions were excluded from analysis.

We partitioned these variants into the following five cMAF ranges: [0–0.001%), [0.001%-0.005%), [0.005% -0.01%), [0.01%-1%), and ≥1%. For each performance metric we report the number of variants over which the metric is computed. For example, positive predictive value can only be computed for a variant where the array has at least one heterozygous or minor homozygous call. Likewise, sensitivity can only be computed when whole exome sequencing data for the variant has at least one heterozygous or minor homozygous call. We report the median and inter-quartile range of the performance metrics since they are not normally distributed. We also report the mean positive predictive value and mean sensitivity for comparison with the results of Weedon et al. [5].

Following the ‘allele match’ variant matching scheme of Krusche et al. [16], we considered any genotype containing the minor allele (i.e., heterozygote or minor homozygote) to be a positive and the major homozygote to be a negative (we considered only bi-allelic variants in this study). This scheme is particularly appropriate in this study because of its focus on rare variants, where the critical event consists of detecting the presence of the rare allele. We note that using the full genotype match scheme (where a mismatch is recorded between a heterozygous and a homozygous minor allele call) had virtually no effect on the mean metrics (difference of no more than 1.4 percentage units) because minor homozygous calls are extremely rare in low MAF variants.

We measured percentage of true positive heterozygotes (TP hets) that were retained after applying RHA as (TP hets post-RHA)/(TP hets pre-RHA). Only heterozygous genotypes are affected by the algorithm.

Missingness in either the reference set or the array data can create a significant bias in the computation of performance metrics. S1 Table summarizes the missingness, yield [17] and effectiveness [18] in the Axiom genotyping (index set), and the percent of missingness in the reference set (stratified by the Axiom genotype call) for the variant groups that were analyzed. Axiom genotyping had over 99% yield and effectiveness in all cases so we made no attempt to account for missing genotypes in the index set. Reference missingness varied significantly by reference set and Axiom genotype and was a potential problem when using 50k FE-VCF. Overall, only 0.4% of Axiom genotypes in reference variants were missing from 200k OQFE-PLINK. However, 6.3% were missing from 50k FE-VCF across all single nucleotide exome variants that passed Axiom quality control filtering. Missingness was lower in the low minor allele ranges, which was the topic of this analysis. Missingness for the four ranges with cMAF<1% was between 3.2% and 4.4% but was 13.4% for cMAF≥1%.

To assess how to handle missing reference genotypes (i.e. unverified genotypes), we calculated and summarized the performance metrics under each of four possible assumptions: 1) complete pair analysis (only consider calls made in both platforms); 2) the ‘missing at random’ (MAR) assumption, which basically states that”No Calls” in the whole exome sequencing data are not biased towards true positives or false positives; 3) all unverified genotypes are negative; and 4) all unverified genotypes are positive (S2 Table) [19]. We compared the performance metric summaries obtained using the 50k FE-VCF reference data to those obtained using 200k OQFE-PLINK data for the same 50k samples and variants in common to both data sets. The comparison was done with each of the four methods (assumptions 1–4) (S3 Table and S1 Fig in S1 File). We proceeded to use the complete-pair method (1) because it produced by far the closest match to the performance metrics for the 200k FE-PLINK data, where missing references were not a significant issue.

A significant focus of this work is the removal of false positive heterozygous calls via the RHA algorithm, and this mainly affects the positive predictive value metric. In the very low minor allele frequency ranges there is a large excess of true negatives, which always produces extremely high specificity. We therefore report only on positive predictive value in the main text, with data for all four performance metrics reported in S2 Table. S4 Table contains the corresponding data for the UK BiLEVE array.

### The Rare Heterozygous Adjusted (RHA) algorithm

The Rare Heterozygous Adjusted (RHA) algorithm is integrated into Axiom™Analysis Suite (AxAS v5.1 and subsequent) and Applied Biosystems™Array Power Tools (APT 2.11.3 and subsequent). It is executed after the original genotyping algorithm (AxiomGT1), to determine which predictions of rare heterozygous genotypes are likely to be false. The genotypes of falsely predicted heterozygotes are then set to “No Call”. The main insight of the algorithm, after examining many hundreds of different array results, is that most falsely predicted heterozygotes occur due to a small number of higher or lower than expected intensities on the microarray surface. Aberrant signals are identified by observing that replicate probes on the array significantly differ in intensity. The AxiomGT1 algorithm clusters samples in signal contrast vs. signal size space (“clustering space”). These unexpected intensities can position the summarized (by median polish) probeset intensities of a homozygous sample in a location that is consistent with a heterozygous genotype, thereby causing the calling algorithm to falsely predict a heterozygote. This is further explained below, after a detailed description of the algorithm.

The AxiomGT1 algorithm used to genotype the UK Biobank already incorporates detection and elimination of unexpected intensities into its genotype calling but does not use higher stringency for variants with low minor allele frequency. RHA improves the prediction of rare heterozygotes by incorporating additional information in the test for unexpected intensities. These include: (1) the intensity differences of replicated probe sequences on the array for the putative heterozygous sample and (2) the position of the signal of the putative heterozygote in relation to the signal distribution of homozygous samples.

**Fig 1:**
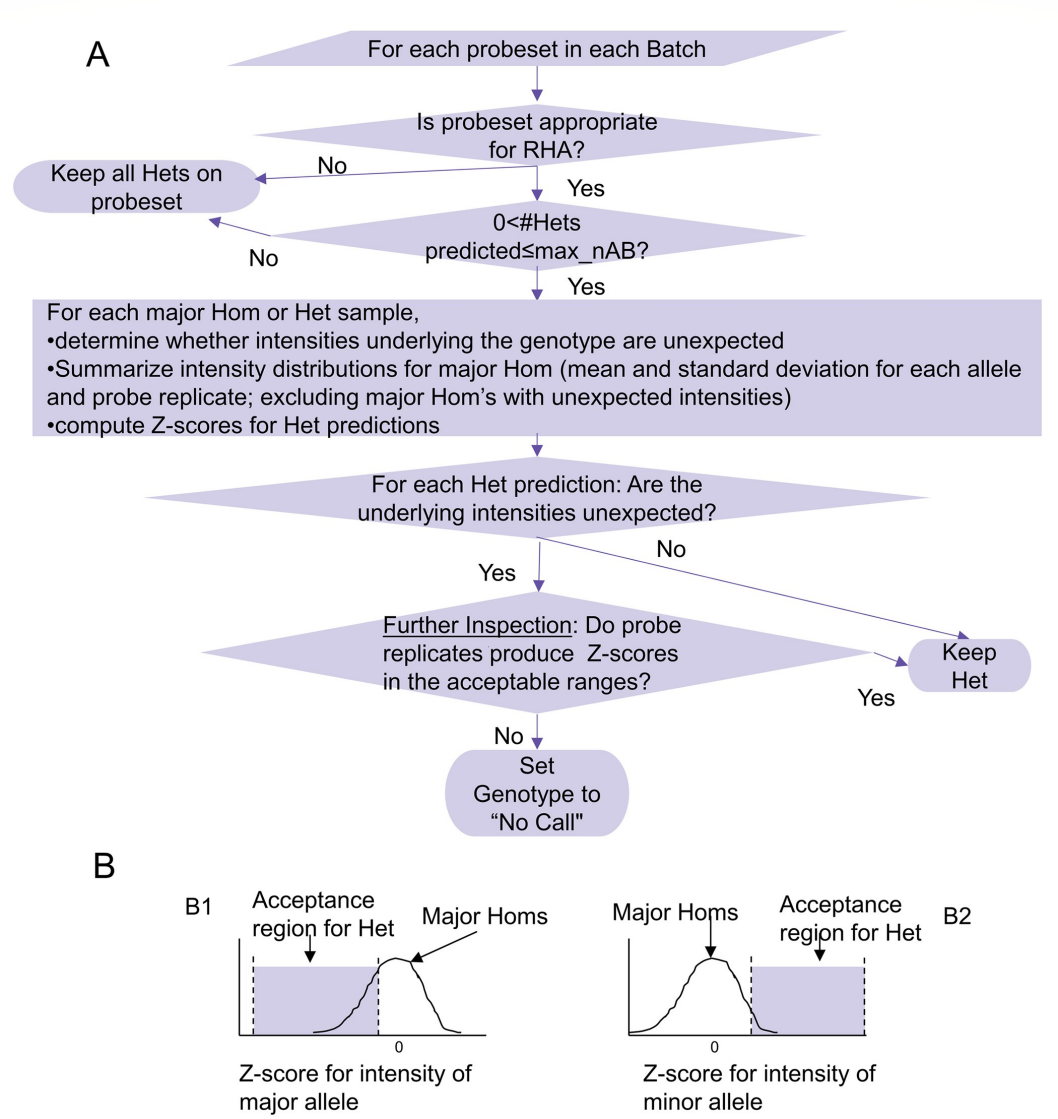
RHA scheme for distinguishing true heterozygotes from false ones. (A) Overall flow of the algorithm. (B) Illustration of acceptable range for heterozygous intensity Z-scores relative to the distribution of intensities from major homozygous samples. (B1) Acceptance region for the Z-score of the major allele intensity. (B2) Acceptance region for the Z-score of the minor allele intensity. Het = heterozygote; Hom = homozygote.

The steps of the RHA algorithm are detailed below and summarized in Fig 1 as a flowchart. It accepts as input the genotypes called by AxiomGT1, the normalized intensities for each sample, and the annotations of locations on the array that were eliminated by AxiomGT1 before calling genotypes.

#### 1. Determine whether the probeset is appropriate for RHA

RHA is only applied to probesets with two replicate probes that measure biallelic variants.

#### 2. Check whether the size of the heterozygous cluster is appropriate for RHA

A configurable parameter, max_nAB, determines the maximum number of heterozygous calls a variant may receive in a given batch in order to be subject to RHA. In the UK Biobank release, samples were genotyped in batches of about 5,000 samples. We applied a max_nAB value of 4; i.e., RHA was applied to variants with cMAF ≤ ~0.04% within the given batch.

#### 3. Mark heterozygous and major homozygous calls that have unexpected underlying intensities

The AxiomGT1 genotyping algorithm clusters samples to obtain genotypes, after summarizing the intensities for the various replicates of the same probe [8]. Different replicates of probe sequences for the same probeset may have slightly different intensity distributions as a result of their locations on the array. Minor non-uniformities on the array surface can cause a probe’s intensity to be lower or higher than it should be.

Calls with unexpected intensities meet one of two criteria:

- **One of the probeset’s probe replicates was eliminated by the Axiom GT1 algorithm because of an unexpected intensity.** For such probes, the genotyping algorithm uses just one of the replicates to genotype the sample. Because the eliminated probe had an unexpected intensity the genotyping call is marked.
- **The difference between intensities measured on different probe replicates is higher than expected.** To predict and classify probe intensities as unexpected, we devised the SCI (Sequence Contrast Index) metric. SCI is calculated separately for the A and B alleles, using I1 and I2, the normalized intensities for the two probe replicates, and can easily be generalized for more replicates:

$$SCI(A)=\frac{abs(I_{1}(A)-I_{2}(A))}{I_{1}(A)+I_{2}(A)};$$ (1)

$$SCI(B)=\frac{abs(I_{1}(B)-I_{2}(B))}{I_{1}(B)+I_{2}(B)}$$ (2)

SCI equals 0 for identical intensities and approaches 1 as the difference between intensities grows. Because both replicates of a probe are unlikely to be affected by the same type of unexpected intensity at the same time, a low SCI indicates that intensities measured on both probe replicates are reliable. A cutoff value for SCI was empirically derived and set to 0.39 based on observations for predicted heterozygous calls and was shown to be applicable for different arrays and batch sizes. The cutoff value for SCI is a configurable parameter of the algorithm.

#### 4. Summarize the intensities of major homozygous calls

For each probeset that is subject to RHA, we compute for each allele and probe replicate the mean and standard deviation of intensities of major homozygous calls. We exclude from this calculation major homozygotes that were marked in step 3 (i.e. had unexpected intensities). In addition, we compute these summary statistics only when, after discarding “No Calls”, at least *90*% of samples are major homozygotes and the number of good quality (i.e. no unexpected intensities) major homozygous genotypes is sufficiently high (at least *30*). Both the minimal percentage of major homozytotes and the minimal number of good quality major homozygous genotypes are configurable parameters of the algorithm.

#### 5. Compute Z-scores for each heterozygous call

For each heterozygous call we use, if available, the summary statistics computed from the major homozygous calls to compute four Z-scores–one for each allele–probe replicate combination. We use the following formula to compute the Z-scores:

$$Z(r,a)=\frac{intensity-mean(majorHomozygousIntensity(r,a))}{stdev(majorHomozygousIntensity(r,a))},$$ (3)

where *r* is the probe replicate (1 or 2) and *a* is the allele (major or minor).

#### 6. Inspect heterozygous calls to determine whether they are likely true

- **Retain heterozygous calls without unexpected intensities.**
- **Further inspect heterozygous calls with unexpected intensities and retain only those for which the Z-scores could be computed (determined in step 4) and are in the acceptable ranges.** For the major allele these correspond to Z-scores in the [-9.25,0] range and for the minor allele these correspond to Z-scores in the [1.5,250] range.

Steps 4–6 bear some elaboration and further explanation. Not all heterozygous predictions with unexpected underlying intensities are false. To minimize loss of true positive heterozygotes, we further examine suspect heterozygous predictions. Ideally, we would compare the intensities driving the heterozygous prediction to a distribution of expected intensities for a heterozygous genotype, as is done for variants with higher minor allele frequency. However, this requires many examples of true heterozygotes for the given variant, which are generally not available for variants with low minor allele frequency. Instead, we compare the predicted heterozygote’s intensities to the distribution of intensities for the major homozygous genotype.

A major homozygous genotype is comprised of two copies of the major allele and zero copies of the minor allele, whereas a heterozygous genotype is comprised of one copy of each allele. These differences are reflected in the intensity measurements of each of the two alleles. The major homozygote intensity distribution differs for different probesets, and sometimes for different replicates. However, we have found that the range of positions relative to this distribution appropriate for a heterozygote is universal for all probesets. We therefore translate the intensities underlying the heterozygous prediction into Z-scores relative to the distribution of intensities of major homozygous genotypes (Eq 3) and check whether these Z-scores fall in the expected or “acceptable” range (Fig 1B). In this way we check for each probe-replicate whether the intensities measured on it are consistent with the genotype call.

The expected range of heterozygote intensity differs for the major and minor alleles (Fig 1B). We expect a heterozygote’s major allele intensity Z-score to be negative because it has only one copy of the allele, versus two copies for a homozygote. However, intensity values and their associated Z-scores that are too low may be indicative of a probe that is not working and are not accepted. We expect the minor allele intensity Z-score to be positive because of the presence of one minor allele, versus none for a major homozygote. On the other hand, a Z-score that is too high could indicate that the intensity measured on the probe is aberrantly high.

If all four Z-scores fall within the appropriate acceptance range, the heterozygote call is retained; otherwise it is set to “No Call.” If we were unable to obtain summary statistics for major homozygotes, all of the probeset’s heterozygotes with unexpected intensities are set to “No Call.”

**Fig 2:**
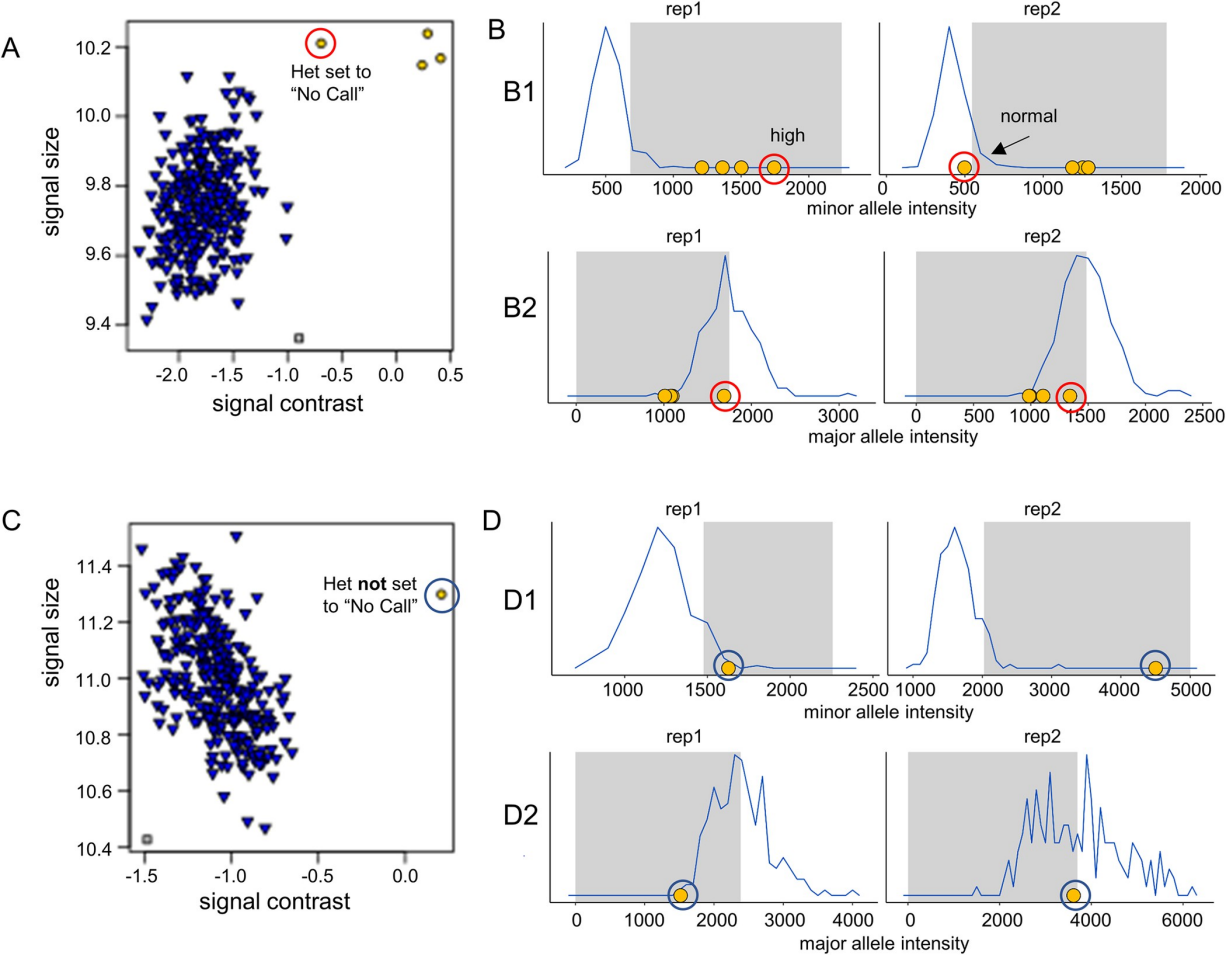
Unexpected intensities can cause false heterozygous calls. (A) & (C): Two examples of cluster plots displaying the summarized intensities of each sample for a given probeset in “signal contrast” vs. “signal strength” space. Each point is the summary (median polish) of two replicate probes (rep1 and rep2) for the same sample. Major homozygous calls are blue upside-down triangles and heterozygous calls are yellow dots. In A one of the heterozygous calls was flagged and is a false positive (circled in red). In C the heterozygous call that was flagged is circled in blue. Data are from Phase II HapMap [20] samples from the NIGMS Human Genetic Cell Repository at the Coriell Institute for Medical Research, representing African, East Asian and European populations. The false positive is in a sample of East Asian origin. All remaining heterozygous calls shown are in samples of European origin. (B) & (D): The blue graphs represent the intensities of good quality major homozygotes in the respective probeset. The shaded grey areas denote the empirically determined intensity ranges where we would expect the intensity of a heterozygote to fall (for better legibility, minor allele expected ranges are truncated on the right). Yellow dots mark the intensities of all samples corresponding to heterozygous calls.

We provide two examples of heterozygous predictions with unexpected underlying intensities in Fig 2 (one spurious call and one correct call).

(B1) Comparison of minor allele intensities. The intensity of the false positive heterozygous call is circled in red. Rep1 has high minor allele intensity (in the grey area), rep2 is not in the grey area. The discrepancy between the two caused the call to be flagged. Because rep2 is not in the (grey) het area, the call is rejected (set to “No Call”).

(B2) Comparison of major allele intensity. Rep2 looks completely within the range of expected intensities for a heterozygote and rep1 does as well; the decision to reject the heterozygous call was already made due to the minor allele intensity of rep1.

(D1) Comparison of minor allele intensities. Both replicates have minor allele intensities consistent with a heterozygous call. Rep2 has a much higher intensity which caused the call to be flagged, but because both reps have high minor allele intensity the heterozygous call is not set to “No Call”.

(D2) Comparison of major allele intensity. Both replicates have intensities in the shaded area (consistent with a true heterozygous call) and the heterozygous call is (in this case correctly) retained.

Note: a flagged heterozygous call is set to “No Call” when at least one of the four panels shows an intensity inconsistent with a heterozygous genotype.

#### Derivation of algorithm parameters

To select appropriate values for RHA parameters, we trained on six independent datasets, each comprising one to three array plates and containing 1000 Genomes Project [21] reference samples. We used sequencing data from the 1000 Genomes Project as truth. Due to the relatively small number of samples in each dataset, training was limited to putative singleton heterozygotes (i.e., max_nAB = 1). Balancing specificity and sensitivity, we used a simple grid search of parameter space to select a cutoff for SCI, and minimum and maximum acceptable Z-scores.

**Fig 3:**
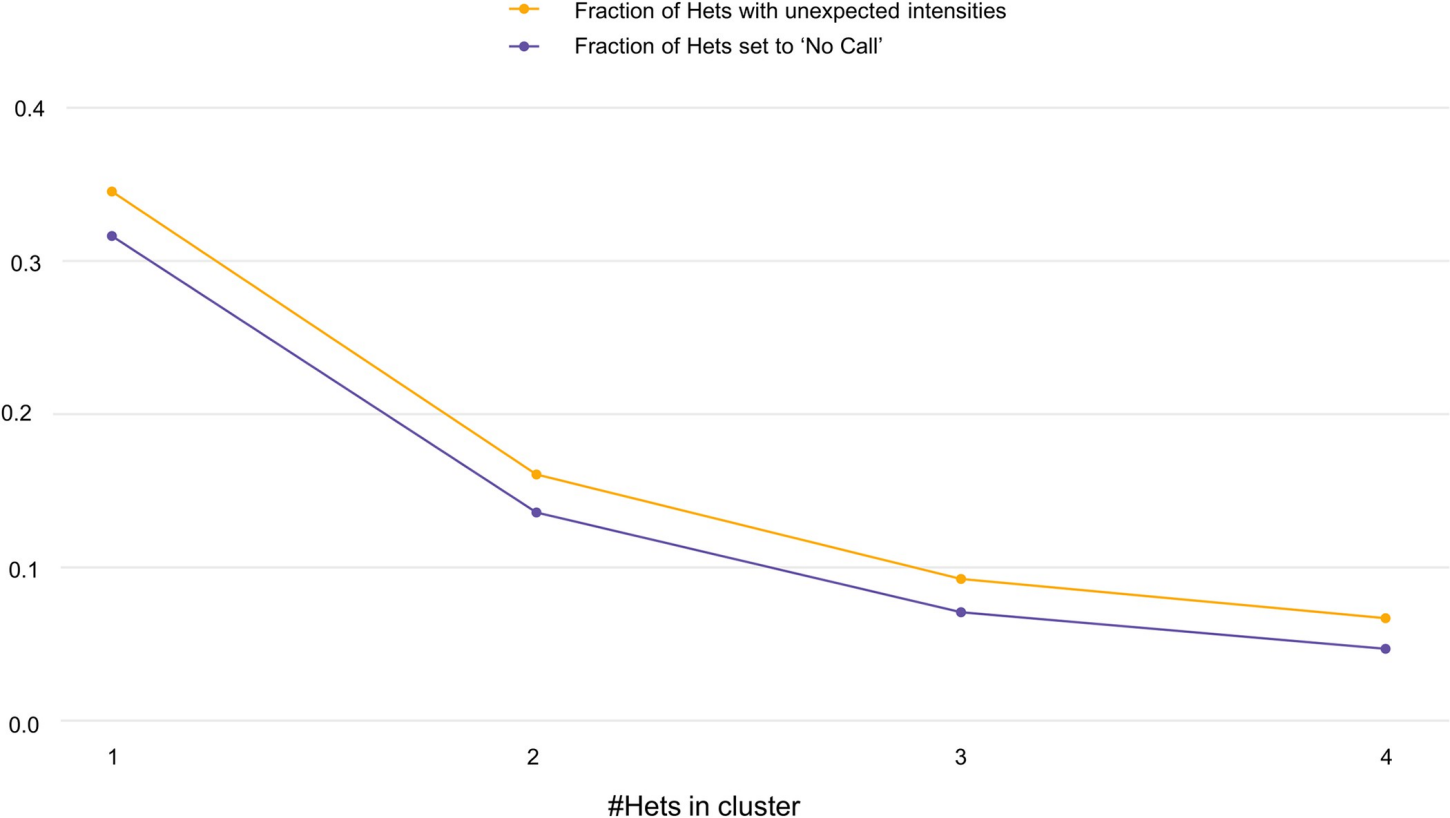
The fraction of predicted heterozygotes that are set to “No Call” by RHA decreases as the size of the heterozygote cluster increases. For cluster sizes ranging from 1 to 4, we show the fraction of predicted heterozygotes with underlying unexpected intensities and the fraction of heterozygotes that are set to “No Call.” Both quantities decrease with increasing size of heterozygote cluster. Het = heterozygote.

In the larger UK Biobank data we found that there was benefit to applying the algorithm with a higher value of max_nAB. We also found that as the number of heterozygous genotypes per cluster increased, fewer heterozygous genotypes had unexpected signals and were set to "No Call” by RHA (Fig 3). While 31.6% of singleton heterozygote calls were set to “No Call” by RHA, only 4.7% of heterozygote calls from heterozygous clusters of size 4 were set to “No Call.” The improvement in positive predictive value afforded by RHA thus plateaued at relatively small values of max_nAB. Balancing specificity with computational cost, we decided to set max_nAB = 4.

## Results

**Fig 4:**
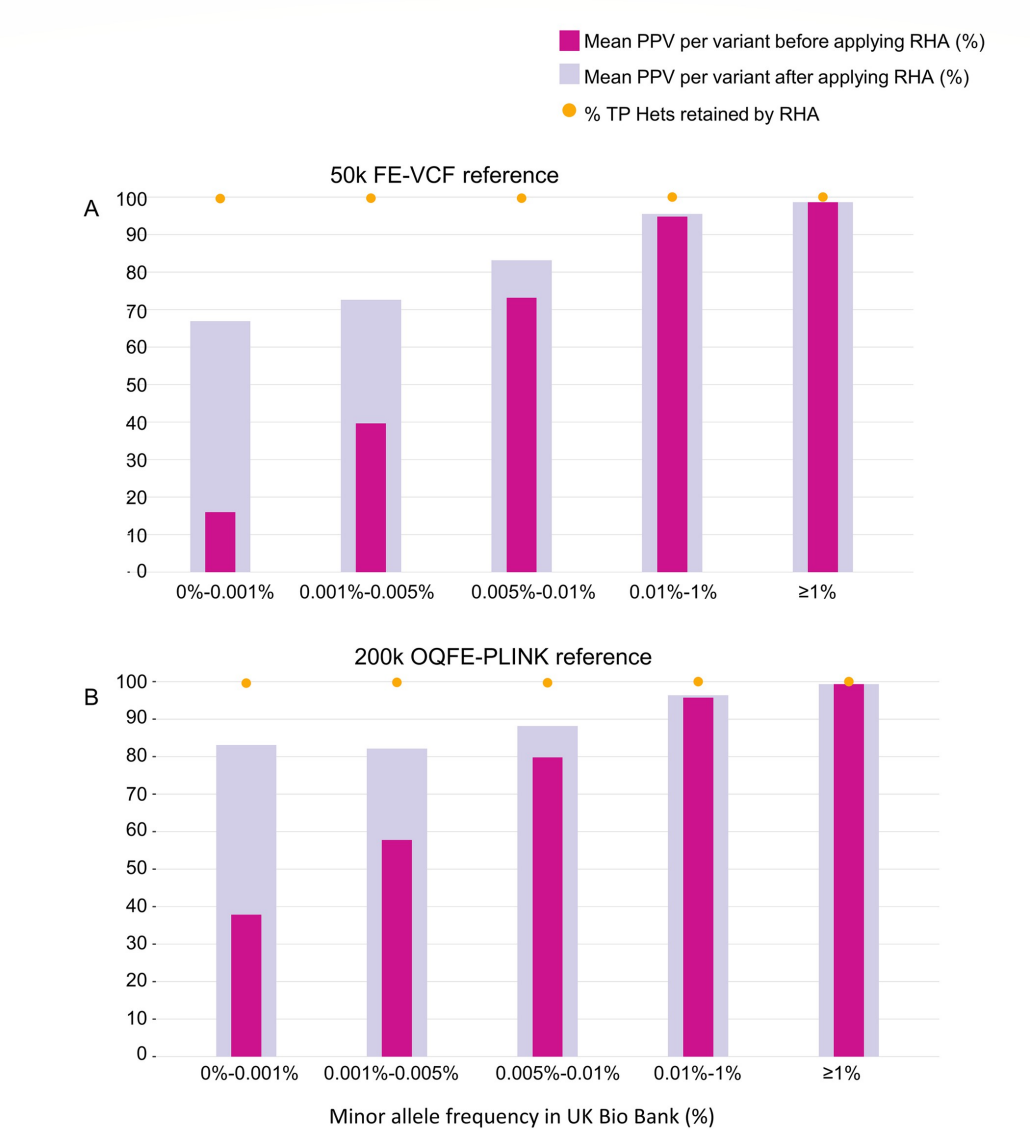
Improvement in positive predictive value of rare variants after application of RHA in the 50k and 200k data. Bars indicate mean positive predictive value (PPV) of variants genotyped by UK Biobank Axiom array. The minor allele frequency ranges were calculated from the genotyping results (cMAF) before applying RHA. We used genotypes from exome datasets 50k FE-VCF (A) or 200k OQFE-PLINK (B) as truth, comparing performance before and after applying RHA. We also indicate the percentage of true positive heterozygous calls (TP hets) retained after applying RHA. Data for the UK BiLEVE Axiom array shows similar trends (S2 Fig in S1 File). Note that for a given cMAF range, the number of variants contributing to the mean may be lower after the application of RHA because for some variants RHA eliminates all heterozygous predictions by the array, so that the positive predictive value cannot be calculated.

We directionally reproduced the positive predictive values obtained by Weedon et al. [5] using the genomic Variant Call Format (gVCF) files for the 50k exome data (50k FE-VCF) restricted to genotype calls covered by more than 15 reads in the sequencing data. Before describing the results, it is helpful to introduce a few notations. Variants monomorphic in the Axiom array data (monoAx) are variants for which the array data calls only the same homozygous genotype for samples in the reference data (50k or 200k). Variants monomorphic in the whole exome sequencing data (monoWES) similarly have only major homozygous genotypes in the respective sequencing data. Results before and after the RHA algorithm showed significant improvements in positive predictive value for low minor allele frequency ranges, on the 50k data (Fig 4A and Table 1) and on the 200k data (Fig 4B and Table 1). To illustrate the effect of the RHA algorithm (irrespective of the reference set) we briefly summarize the results using the 50k data as reference. In the lowest cMAF range (below 0.001%) the mean of the per-variant positive predictive values improved from 16% to 67%. This lowest cMAF range contained fewer than 5,000 array heterozygous genotypes across only 3,600 variants. For cMAF ranging between 0.005%-0.01% the mean positive predictive value increased to at least 83%, while for cMAF ranging between 0.01%-1% the mean positive predictive value increased to 95.5%.

**Table 1:** Positive predictive value of UK Biobank Axiom™ array versus whole exome sequencing before and after application of RHA.

Reference and dataset	Before/ After application of RHA	Positive Predictive Value (%)			%TP hets retained after RHA
		Number of variants	Median (interquartile range)	Mean	
**50k FE-VCF (n = 45,895)**					
**All exome variants**	Before	85,447	99.7% (95.0%-100.0%)	84.5%	
	After	78,468	99.9% (98.4%-100.0%)	**93.6%**	>99.9%
**cMAF 0%-0.001%**	Before	3,568	0.0% (0.0%- 0.0%)	16.0%	
	After	934	100.0% (0.0%-100.0%)	**66.9%**	99.6%
**cMAF 0.001%-0.005%**	Before	11,134	0.0% (0.0%-100.0%)	39.6%	
	After	6,968	100.0% (50.0%-100.0%)	**72.6%**	99.7%
**cMAF 0.005%-0.01%**	Before	4,107	90.9% (57.1%-100.0%)	73.2%	
	After	3,954	100.0% (83.3%-100.0%)	**83.2%**	99.7%
**cMAF 0.01%-1%**	Before	38,980	99.2% (96.3%-100.0%)	94.8%	
	After	38,954	99.4% (96.9%-100.0%)	**95.5%**	>99.9%
**cMAF≥1%**	Before	27,658	99.9% (99.6%-100.0%)	98.6%	
	After	27,658	99.9% (99.6%-100.0%)	98.6%	100.0%
**200k OQFE-PLINK (n = 195,447)**					
**All exome variants**	Before	85,591	99.4% (95.7%- 99.9%)	89.8%	
	After	83,647	99.7% (98.2%-100.0%)	**95.2%**	>99.9%
**cMAF 0%-0.001%**	Before	3,358	33.3% (0.0%- 66.7%)	37.9%	
	After	2,135	100.0% (100.0%-100.0%)	**83.1%**	99.6%
**cMAF 0.001%-0.005%**	Before	9,358	66.7% (22.2%- 93.3%)	57.7%	
	After	8,642	100.0% (75.0%-100.0%)	**82.2%**	99.8%
**cMAF 0.005%-0.01%**	Before	3,961	91.3% (72.7%-100.0%)	79.8%	
	After	3,956	100.0% (88.9%-100.0%)	**88.2%**	99.7%
**cMAF 0.01%-1%**	Before	40,020	98.9% (96.5%- 99.8%)	95.7%	
	After	40,020	99.1% (97.1%- 99.9%)	**96.4%**	>99.9%
**cMAF ≥1%**	Before	28,894	99.9% (99.6%-100.0%)	99.3%	
	After	28,894	99.9% (99.6%-100.0%)	**99.3%**	100.0%

Results are split by the whole exome sequencing dataset used as reference and by the minor allele frequency calculated from the array before application of RHA (cMAF). We include the following summary statistics of the distribution of positive predictive values, calculated across all relevant single nucleotide variants: median, mean, and inter-quartile range. “Number of variants” is the number of variants the summary statistics have been calculated over. We also include “%TP hets retained after RHA”, which indicates the small drop in sensitivity imposed by the RHA algorithm.

The differences in mean positive predictive value (pre-RHA) between Fig 4A and 4B, also seen in Table 1, arise mostly from the effect of monoWES variants for which the array data produces a few heterozygous calls. There are monoWES loci in the 50k FE-VCF calls but not in the 200k OQFE-PLINK data. The difference significantly diminishes post-RHA, since RHA detected and removed most false heterozygous calls in these and other variants.

The three lowest frequency groups ([0,0.001%), [0.001%, 0.005%) and [0.005%, 0.01%)) showed an initial mean positive predictive value of 38%, 58% and 80% and improved to 83%, 82% and 88%, respectively. Variants with cMAF of at least 0.01% but less than 1% and variants with a cMAF of at least 1% had an initial mean positive predictive value of 95% and 99%, respectively, and only marginally benefitted from the new algorithm.

**Fig 5:**
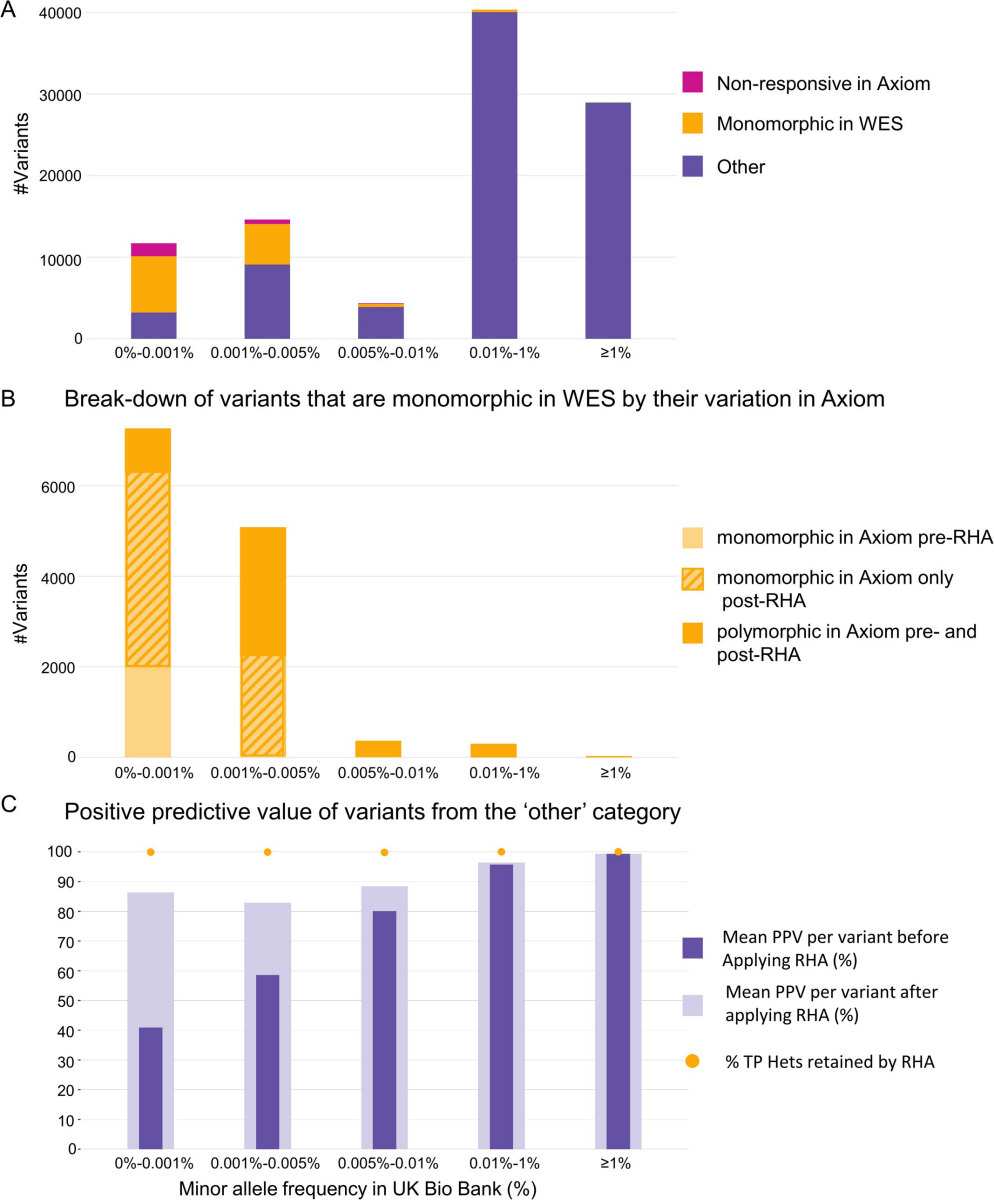
At very low cMAF (≤0.005%) a large proportion of variants are monomorphic in the exome sequencing (monoWES). (A) The variants in various cMAF ranges are divided into three groups. Group 1 is non-responsive in array data (“non-responsive in Axiom”). Group 2 is monoWES (“monomorphic in WES”). Group 3 includes all remaining variants (“other”). Note that a variant can be both non-responsive in the array data and monoWES; in this panel such variants are counted in the “non-responsive in Axiom” category. Variants that are monoWES make up 59% and 34% of the total variants in cMAF ranges 0%-0.001% and 0.001%-0.005%, respectively. (B) Further partitioning of monoWES variants into three subgroups. The partitioning is of all 13,049 monoWES variants, including those labeled as monoWES in Group 1 as well as the 464 probesets that are identified as non-responsive (included in Group 1 bars in A) but are also monoWES. 2a: monoAx pre-RHA (2,025 variants); 2b: monoAx only post-RHA (6,518 variants); 2c: polymorphic in Axiom pre- and post-RHA (4,506 variants). Bars indicate the total number of group 2 variants, as well as the proportion of each subgroup, for each cMAF range. (C) Mean positive predictive value (PPV) of variants genotyped by UK Biobank Axiom array, using genotypes from 200k OQFE-PLINK as reference, and restricting to variants that are polymorphic in the exome sequencing and responsive in the array data. Bars indicate PPV before and after applying RHA; dots indicate the percentage of true positive heterozygous calls retained after applying RHA.

To elucidate both the content in the various minor allele frequency ranges, and the effect of having no monoWES variants in the reference data, we divided the variants in each minor allele frequency range into three groups (Fig 5A and S5 Table).

### a. Non-responsive in Axiom (Group 1)

This group is defined as variants that have low minor allele frequency in the array data but have high minor allele frequency in the population, based on other evidence. These correspond to non-responsive probesets in the array data. While variants in this group generally have reasonable positive predictive value, they have very poor sensitivity and should be filtered from the array data. The number of such variants is small but they have significant deleterious effects on the overall sensitivity of the array data. We defined a variant as non-responsive if the minor allele frequency of the variant in the Non-Finnish European (NFE) population in the Genome Aggregation Database (gnomAD) [22] was significantly higher than its cMAF. Specifically, the variant is considered non-responsive if gnomAD minor allele frequency is > = 10*M_r_, where M_r_ equals the upper bound of the range of cMAF the variant falls into, i.e. [0, 0.001%), [0.001%, 0.005%), [0.005%, 0.01%), [0.01%, 1%). Table 2 shows the positive effect on sensitivity of excluding non-responsive probesets.

**Table 2:** Effect of exclusion of probesets non-responsive in array on post-RHA sensitivity.

cMAF range	Before/After exclusion of non-responsive probesets	Sensitivity (%)		
		Number of variants	Median (interquartile range)	Mean
**cMAF 0%-0.001%**	Before	3,329	33.3% (0.0%-100.0%)	49.5%
	After	2,214	100.0% (0.0%-100.0%)	**70.1%**
**cMAF 0.001%-0.005%**	Before	8,756	100.0% (100.0%-100.0%)	84.1%
	After	8,328	100.0% (100.0%-100.0%)	**87.8%**
**cMAF 0.005%-0.01%**	Before	3,943	100.0% (100.0%-100.0%)	92.8%
	After	3,864	100.0% (100.0%-100.0%)	**94.3%**
**cMAF 0.01%-1%**	Before	40,007	100.0% (100.0%-100.0%)	98.4%
	After	40,006	100.0% (100.0%-100.0%)	**98.4%**
**cMAF ≥1%**	Before	28,893	100.0% (100.0%-100.0%)	99.9%
	After	28,893	100.0% (100.0%-100.0%)	**99.9%**

Results were obtained using the 200k OQFE-PLINK reference and are split by the minor allele frequency calculated from the array (cMAF) before application of RHA. We include the following summary statistics for the distribution of sensitivity values, calculated across all relevant single nucleotide variants: mean, median and inter-quartile range. We also include the number of variants over which the summary statistics have been calculated.

### b. Variants that are monoWES (Group 2: “Monomorphic in WES”)

This group always has a positive predictive value of 0 for all variants with a heterozygous call in the array data, since, by definition, there are no true positive heterozygotes in this group. It has the largest improvement post-RHA in eliminating spurious heterozygotes. In some sense, while this group can contain ultra-rare variants of interest, the majority of these correspond to loci in the genome that are missing entirely from gnomAD [22] and are likely homozygous in any population of similar ancestry and size. These were included in the array design based on early sequencing results but have proven to be fundamentally not informative in the UK Biobank cohort, since none of the 200k participants for which exome sequencing data is available carry the minor allele.

To understand the contribution of monoWES variants to the positive predictive value we further break them into three subgroups, corresponding to their polymorphic status in the UK Biobank array data (Fig 5B and Table 3). The three subgroups are: 2a, “monoAx pre-RHA”; 2b, “monoAx only post-RHA”; 2c, “polymorphic in Axiom pre- and post-RHA”. Polymorphic status pertains only to the 200k exome-sequenced samples. The third subgroup is the only one that remains non-concordant with monoWES variants post-RHA.

**Table 3:** Polymorphic status in Axiom UK Biobank array of Group 2 single nucleotide variants that are monomorphic in the 200k whole exome sequencing data.

cMAF range	Number of monoWES variants	Number of monoWES variants monomorphic in Axiom pre-RHA (2a)	Number of monoWES variants monomorphic in Axiom only post-RHA (2b)	Number of monoWES variants polymorphic in Axiom pre- and post-RHA (2c)
**0%-0.001%**	7,267	1,982 (27%)	4,315 (59%)	970 (13%)
**0.001%-0.005%**	5,082	42 (0.8%)	2,189 (43%)	2,851 (56%)
**0.005%-0.01%**	368	1 (0.3%)	13 (4%)	354 (96%)
**0.01%-1%**	303	0	1 (0.3%)	302 (99.7%)
**≥1%**	29	0	0	29 (100%)
**≥0% (total)**	13,049	2,025 (16%)	6,518 (50%)	4,506 (35%)

Variants are divided into three categories: “monomorphic in Axiom pre-RHA (2a)”, “monomorphic in Axiom only post-RHA (2b)” and “polymorphic in Axiom pre- and post-RHA (2c)”. Note that for the purpose of this classification we consider for each variant only samples that have a genotype in both Axiom and whole exome sequencing, so that a variant can be both monomorphic in Axiom pre-RHA and have cMAF > 0.

In the lowest cMAF range (<0.001%), there are 7,267 monoWES variants, 27% of these (1,982) are also monoAx and do not produce any false positives in the array even without RHA. Applying RHA eliminates almost 90% of false heterozygous predictions in the remaining 73% of these variants, leaving only 970 (13%) of these variants to produce a total of 1,220 heterozygous predictions not in the sequencing data.

Overall, there are 13,049 monoWES variants. 2,025 are also monoAx pre RHA and 8,543 are monoAX post RHA, (Table 3).

For cMAF ranges 0.001% to 0.005% and 0.005% to 0.01%, while a significant proportion of the monoWES variants remain polymorphic in the array post-RHA, (56% and 96%, respectively), most false positive heterozygotes in this variant class are eliminated by RHA (77% and 60%, respectively). For cMAF between 0.01% and 1% RHA has little effect on heterozygous predictions because RHA is only applied to small heterozygote clusters, which are rarely found in this minor allele frequency range.

### c. All other variants (Group 3)

These variants are most informative for the computation of positive predictive value and along with non-responsive variants (Group 1) are the only ones that contribute to sensitivity.

### Variants in which RHA eliminated all heterozygous calls tend to have extremely low NFE MAF in GnomAD

There are 6,469 probesets where RHA eliminated all heterozygous calls. To evaluate whether these adjustments are reasonable (without the use of whole exome sequencing reference data) we compared them to expected allele frequencies in GnomAD for the non-Finnish European (NFE) population:

- 9% (572) have been identified as non-responsive on the array even before applying RHA.
- 91% (5,897) essentially agree post-RHA with the GnomAD expected NFE frequency:
  ○ 69% (4,444) were either absent or had NFE MAF of 0 in GnomAD
  ○ 22% (1,453) have an average NFE MAF of 2.8e-05 in GnomAD.

### Changes in positive predictive value resulting from increased quality control of exome sequencing

**Fig 6:**
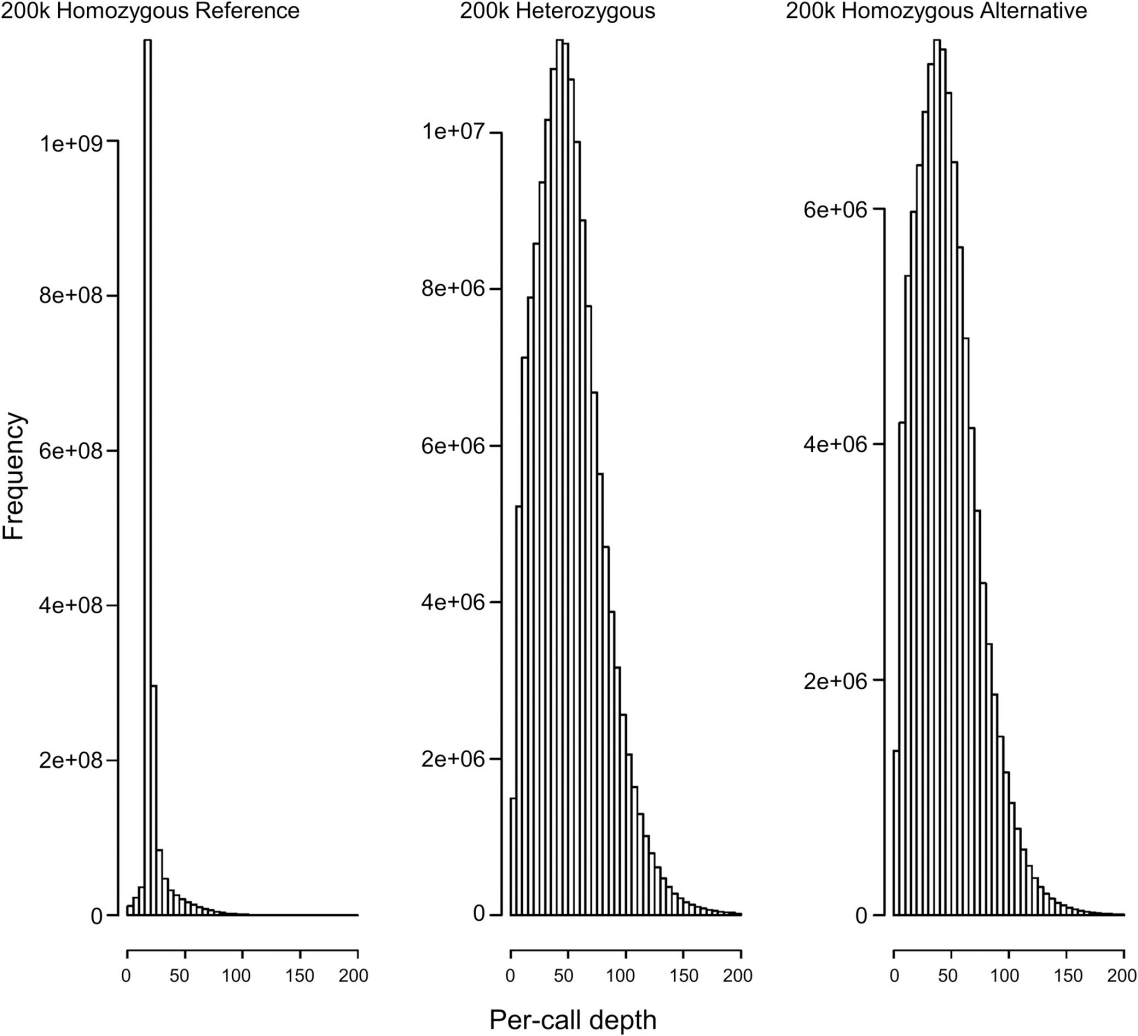
Comparison of sequencing depth by exome genotyping call for the 200k OQFE-PLINK whole exome sequencing data set. Sequencing depth for exome reference homozygous calls, which tend to correspond to major homozygous calls, is significantly lower than sequencing depth for exome heterozygous calls.

Since the 200k whole exome sequencing data was not filtered prior to release [2], we examined the effect of more stringent quality control on our results. We tested a range of cutoff values for sequencing depth. Restricting sequencing data to read depth of at least 5 or 15 had only small effect on positive predictive value. However, imposing a cutoff of 20 reads led to a dramatic increase in mean positive predictive value. For example, in the lowest cMAF range, imposing depth cutoffs of 0 (no filtering), 5, 15, and 20 produced post-RHA mean PPV of 83.1%, 83.1%, 83.3% and 90.4% respectively. This is because most (61%) major homozygous sequencing calls have depth less than 20 (Fig 6), whereas most (86%) heterozygous calls have depth greater than 20. Filtering at this level disproportionately removes the reference for array false positives, artificially improving PPV. We therefore filtered with a modest depth cutoff of 5 to avoid introducing a bias and do not report results from higher cutoffs.

### Probeset filtering

Probesets can be filtered in many ways, most notably by pre-qualifying probesets based on genotyping results for known samples. In the current analysis the only filtering we applied was the most obvious one: eliminating non-responsive probesets for which the known minor allele frequency is significantly higher than the computed minor allele frequency in the array data (Table 2 and S7 Table).

### Variants in the *BRCA* genes

Weedon et al. [5] identified 1,139 variants of interest in *BRCA1* and *BRCA2*. Only 91 of these variants are present on the UK Biobank array, while 1,089 are on the UK BiLEVE array. Only 8% of the 50k whole exome sequencing cohort, and only 3% of the 200k whole exome sequencing cohort, have data from the UK BiLEVE array.

After manually examining the whole exome sequencing data, Weedon et al found that 425 of the 1,139 variants had at least one array heterozygous call that could be compared to whole exome sequencing data [5]. Of these 425 variants, only 53 are present on the UK Biobank array; the remaining 372 are only on the UK BiLEVE array.

Both arrays did indeed have a significant number of false positives. In the 50k whole exome sequencing cohort, the 53 *BRCA* variants with array positives on the UK Biobank array yielded a total of 189 array heterozygotes, of which 157 were not reproduced by whole exome sequencing. While 16 of these 157 were “No Calls” in the 50k FE-VCF data, the corresponding variants are monomorphic in the 200k whole exome sequencing data and we therefore added them to the false positive count. Thus, in agreement with Weedon et al. [5] the overall pre-RHA positive predictive value is 16.9%. RHA eliminated 112 (71.3%) of these false positives while preserving all true positives, thus improving the positive predictive value to 41.6%.

Weedon et al. [5] also identified 65 individuals with one false negative each among the UK Biobank participants genotyped on the UK Biobank array, but 60 of these presumed false negatives were UK BiLEVE-only variants that were not actually assayed on the UK Biobank array. The reported low sensitivity of the array appears to be the result of Weedon et al. assuming these variants were present on both arrays. When considering only variants that are present on the UK Biobank array, and only participants with genotypes from that array, we find 37 sequencing heterozygous calls in the 50k whole exome sequencing cohort (two of the 37 were “No Call” in the filtered 50k FE-VCF but were heterozygotes in the 200k OQFE-PLINK dataset). 32 of these sequencing heterozygous calls were also called heterozygous on the UK Biobank array. Of the five extra heterozygous calls in whole exome sequencing (across five individuals) three received an array “No Call”, leaving two false negatives for an overall sensitivity of 94.1%. The remaining 60 individuals did indeed have a heterozygous call in whole exome sequencing in one of the *BRCA* variants of interest, but they were on variants not present on the UK Biobank array. The large number of *BRCA* variants only present on the UK BiLEVE array underscores the exploratory nature of the probesets for these *BRCA* variants.

We further note that the UK Biobank array has 460 *BRCA* variants with cMAF < 0.01% that are included in the 200k OQFE-PLINK data set. Pre-RHA these include 4,739 positives called by the array, 2,982 positives called by exome sequencing and 2,528 called by both, for a pre-RHA sensitivity of 84.8% and positive predictive value of 53.3%. RHA removed 1,613 (73%) of the false positives and three (0.1%) true positives, increasing the overall positive predictive value to 80.9% while reducing the sensitivity to 84.7%.

Table 4 and S8 Table contain additional summary statistics for *BRCA* performance on the UK Biobank and UK BiLEVE arrays.

**Table 4:** Performance of Axiom UK Biobank array on the BRCA module.

	50k Exome data (FE-VCF)	200k Exome data (OQFE-PLINK)
**Variants**	91	26
**True Positive**	32	141
**False Positive**	157	199
**False Negative**	2	12
**Exome Het**	3	21
**Array NC**		
**Sensitivity**	94.1%	92.2%
**PPV (Pre-RHA)**	16.9%	41.5%
**PPV (Post-RHA)**	41.6%	65.3%

Whole exome sequencing data from 50k FE-VCF or 200k OQFE-PLINK is used as a reference. The variants included are those studied by Weedon et al. [5]. Of the 91 variants present on the UK Biobank array, 53 variants were included in the list of variants with array heterozygotes or sequencing heterozygotes that Weedon et al. used in their analysis and shared with us. The analysis using the 200k whole exome sequencing release as reference follows these same variants but excludes variants that are monoWES and hence missing in the 200k OQFE-PLINK data. Each data point represents one pair of genotyping calls on one sample. For the 50k FE-VCF release, all variant-sample pairs with a heterozygous call from the array and a “No Call” from sequencing are assigned as true positive or false positive based on the 200k OQFE-PLINK data. Cases where the variant was missing from 200k OQFE-PLINK and was monomorphic in 50k FE-VCF are considered false positives. For the 200k whole exome sequencing release, instances where one of the two technologies was a “No Call” are excluded from the counts. Het = heterozygote; NC =“No Call”; PPV = positive predictive value.

## Discussion

### Statement of principal findings

We have shown that the addition of improved algorithms to the Axiom genotyping pipeline greatly decreases the number of false positive heterozygote calls for rare variants without affecting true positive calls. We also used the power of multiple, large sequencing projects, collected in the Genome Aggregation Database (gnomAD) [22], to filter variants by expected allele frequencies. Filtering out non-responsive probesets in this way detected non-working probesets that should be removed from the array as well as led to significantly higher and more realistic sensitivity values for the detection of ultra-rare variants. This operation was less possible and less accurate at the time of the original calling.

For the variants in the *BRCA* genes, we have found a much higher sensitivity than was found by Weedon et al. [5], because they did not distinguish between sequencing positives that were array negatives, those with missing information because of “No Calls” on the array, and missing information because some variants were not present on a given array. We have also shown that while many variants perform well, a few poorly performing probesets can significantly affect the overall positive predictive value. In addition, a large number of variants with poor performance in the *BRCA* genes were only present on the UK BiLEVE array (used to genotype the first 50k individuals). That array also provided opportunity for additional quality control. Indeed, many of these variants were subsequently excluded from the UK Biobank design.

### Strengths and weaknesses of the study

In this study we compared the two genotyping datasets available at the time of our analysis for the UK Biobank cohort, using genotypes from whole exome sequencing as the reference set and Axiom microarray genotypes as the index set. We used the 200k whole exome sequencing data for our primary result, while using the earlier 50k release for comparison to Weedon et al. [5]. The high accuracy of genotypes based on whole exome sequencing [14, 23], the very large number of variants and genotypes included in the comparison, and the independence of the two genotyping methods give credence to the results presented here.

On the other hand, assuming that the exome sequencing reference is perfectly accurate introduces a bias, which might result in underestimating array performance. Our data show that some variants have very poor performance, but for most variants, Axiom genotyping with the addition of RHA has excellent concordance with the whole exome sequencing data. We can conservatively assume that when these two very different genotyping platforms have high concordance on a variant across a very large number of samples then both are correctly genotyping the variant. When there is poor concordance on a variant, we must assume that at least one platform is wrong. In labeling the whole exome sequencing genotyping as reference, we have implicitly assumed that all errors are in the index (microarray) dataset. However, while whole exome sequencing genotypes are highly accurate they do not have perfect performance either at the level of single sample genotyping [14] or at the level of genotypes derived from the joint genotyping of the samples in the population [23]. In addition, we have chosen to apply only modest quality control to the 200k exome sequencing because application of measures such as read depth filtering disproportionately affects certain call types. Therefore, our estimates of microarray genotyping performance on the UK Biobank dataset is a slight underestimate of the true performance of this platform.

### Strengths and weaknesses in relation to other studies

Several studies evaluated Axiom genotyping on very large numbers of samples prior to the addition of the improved genotyping algorithms and found evidence that genotyping performance deteriorates as minor allele frequency decreases [1, 5, 24, 25]. In three of these studies [1, 24, 25] reference genotypes were not available and the relationship between probeset performance and minor allele frequency was observed through indirect measures: probeset quality control measures [1, 24] and the concordance in genotypes between intentional duplicate samples [24, 25]. Our comparison is against a very large reference and provides a direct and robust evaluation of performance.

Weedon et al. [5] compared Axiom genotyping to whole exome sequencing in the UK Biobank cohort, showing that positive predictive value decreases with minor allele frequency. However, their analysis was limited by the fact that at the time of their analysis only the initial release of whole exome sequencing data was available, covering ~10% of this cohort. We recapitulated the results of Weedon et al. on the same dataset and expanded the analysis to the later 200k whole exome sequencing release [4]. The results showed that A) the same trends exist in the expanded dataset, and B) that the improvements to the Axiom genotyping algorithms significantly improve performance when compared to either. In addition, in their analysis of *BRCA* variants, Weedon et al. introduced a significant bias. Samples that were genotyped on the UK Biobank array and had a positive exome sequencing call were counted as false negatives even if the variant was present only on the UK BiLEVE array, not on the UK Biobank array. This significantly underestimated the sensitivity of the UK Biobank array. In addition, “No Calls” on either array were assumed to be negatives, further decreasing the estimated sensitivity. Thus, both un-assayed variants and inconclusive calls were interpreted as false negatives. We removed such cases from our sensitivity calculation and therefore observed a much higher and more accurate estimate of sensitivity on the *BRCA* variants.

### Meaning of the study

Ultra-rare variants are increasingly included on genotyping microarrays [24, 26–29]. Since microarrays are not generally suitable for discovery of novel polymorphisms, these rare variants are mostly known from the literature or public databases [12, 30], and have previously established or suspected significance. Rare variants are frequently included for research purposes on arrays, which are indeed Research Use Only (RUO), and raw results should not be returned to naïve test subjects or customers of direct-to-consumer genetics companies [31]. Relevant to the main research use of these arrays, statistical power to detect associations to ultra-rare variants in non-case-control studies is limited. Sporadic false positives or negatives, at a low rate that would leave the power of more common variants essentially unchanged, could have a much greater effect on the analysis of ultra-rare variants.

We have shown that microarrays can be used for correctly genotyping ultra-rare variants provided that

1. the probeset used for genotyping has been shown to be responsive to the minor allele; and
2. improved algorithms are used to eliminate spurious heterozygous calls.

Weedon et al. state that because genotyping arrays typically assay many thousands of rare variants simultaneously, and have a specificity that is less than 100%, false positive results will occur and outnumber true positives across all rare variants [5]. This no longer holds after the introduction of the new RHA genotyping algorithm. Our results show a mean positive predictive value above 80% in all minor allele frequency ranges and near or above 90% in minor allele frequency ranges above 0.005%, and a median positive predictive value that is near 100% for all minor allele frequency ranges.

Should the UK Biobank re-release array genotypes based on this work it could improve the results of many research efforts focused on rare variants.

### Future research

Applying the RHA algorithm removes most of the prediction noise in rare variants. Careful analysis shows that some probesets genotype poorly even after applying our improved algorithms. Using the expanding genomic data in the UK Biobank will allow us to detect the underlying reasons for such performance and improve the design of future probes.

## Conclusion

The UK Biobank Axiom array is a research use only array and has provided the community with a large number of insightful, novel, and consequential research results (https://www.ukbiobank.ac.uk/enable-your-research/publications). The new calling algorithm and probeset filtering described here further and significantly improve the accuracy of the resulting data, show high concordance to whole exome sequencing data, and provide an important resource for the research community. Weedon et al. also conclude that clinicians should validate array results from direct-to-consumer companies or research biobanks by using a standard diagnostic test before recommending any action [5]. These recommendations apply to and should be standard practice for all Research Use Only products, including the whole exome sequencing UK Biobank data.

### Glossary

Allele: each of two or more alternative forms of DNA that are found at the same location on a chromosome

Allele A and allele B: For a SNP or indel the two alternatives that can be observed and measured in a given sample are designated as “allele A” and “allele B”

Array: DNA microarray that is used to genotype known genetic variants (SNPs and indels) in the population

Clustering space: The X and Y dimensions defined by Signal Contrast and Signal Size

Effectiveness: proportion of reference genotypes that have a concordant genotype call in the index set ((true positive+ true negative) / reference genotypes)

Exome: ~1–2% of the human genome that codes for proteins

Genotyping: method for determining the alleles present at a specific location in a person’s DNA

Heterozygous (adjective) or heterozygote (noun): two different alleles at a given locus in an individual

Homozygous (adjective) or homozygote (noun): two identical alleles at a given locus in an individual

Indel: type of variant where one or more bases are inserted or deleted as compared to the reference genome

MAF: Minor allele frequency, the allele frequency of the less common allele in a bi-allelic variant

cMAF: Minor allele frequency computed from the array genotypes in the UK Biobank data. Specifically, cMAF is computed for any variant on the array as (number of minor alleles called in the array data)/(number of total alleles called in the array data).

Major Homozygous: two identical alleles at a given locus that represent the most common allele at that locus for the population of interest

Minor Homozygous: two identical alleles at a given locus that represent the less common allele at that locus for the population of interest

Mean positive predictive value over a set of variants and individuals: the average over the positive predictive values for each variant

Missingness: the proportion of calls that are missing either due to being declared as a “No Call” by the genotyping algorithm or due to being filtered out during quality control.

nAB: the number of samples called heterozygous by the AxiomGT1 calling algorithm

Negative predictive value: proportion of the normal alleles that are confirmed by the reference standard (true negative / (true negative + false negative))

Positive predictive value: proportion of variant alleles that are confirmed by the reference standard (true positive / (true positive + false positive))

Overall positive predictive value: Positive predictive value across all variants and samples

Probeset: A specific set of DNA oligomer probes on the microarray that detect the presence of specific alleles at a given locus

Sensitivity: proportion of variant alleles detected by the reference standard that are also found by the index test (true positive / (true positive + false negative))

Signal Contrast and Signal Size: AxiomGT1 genotype clustering is carried out in two dimensions. The X dimension is called “contrast” and the Y dimension is called “size”. They are log-linear combinations of the two normalized allele signal intensities. For alleles A and B, contrast is log2(A/B) and size is (log2(A) +log2(B))/2

Single nucleotide polymorphism (SNP): type of single nucleotide variant; a position in the genome where an individual differs from the reference human genome by a single base change (i.e., a substitution of a single letter of DNA). A SNP may be rare or common in the population

Specificity: proportion of normal alleles detected by the reference standard that are also found to be normal by the index test (true negative / (true negative + false positive))

Variant: Locus in the genome where an individual’s genome differs from the reference human genome

Yield: proportion of genotypes with reference call that also have a call in the index set ((true positive + false positive + true negative + false negative)/reference calls)

## Supporting information

S1 File Supplementary materials for Novel genotyping algorithms for rare variants significantly improve the accuracy of Applied Biosystems™ Axiom™ array genotyping calls: Retrospective evaluation of UK Biobank array data (S1 and S2 Figs). (DOCX)

S1 Table Missingness in the UK Biobank Axiom array (index set) and whole exome sequencing (reference set) data. (XLSX)

S2 Table Summary of UK Biobank Axiom array performance versus whole exome sequencing before and after applying RHA. (XLSX)

S3 Table Comparison of UK Biobank Axiom array performance using the 50k FE-VCF data as reference to the performance on the same samples using the 200k OQFE-PLINK data as reference. (XLSX)

S4 Table Summary of UK BiLEVE Axiom array performance versus whole exome sequencing before and after applying RHA. (XLSX)

S5 Table Distributions of single nucleotide variants with coverage by both Axiom UK Biobank array and whole exome sequencing (WES) among the three Groups. (DOCX)

S6 Table Effect of adding monoWES probesets on the positive predictive value of UK Biobank Axiom array. (DOCX)

S7 Table Summary of UK Biobank Axiom array performance versus whole exome sequencing before and after applying RHA, restricting to variants polymorphic in sequencing and responsive on the Axiom array. (XLSX)

S8 Table Characterization of BRCA variant performance on the Axiom™ arrays used by UK Biobank. (DOCX)
