# Supplementary material for: Novel genotyping algorithms for rare variants significantly improve the accuracy of Applied Biosystems™ Axiom™ array genotyping calls: Retrospective evaluation of UK Biobank array data
Source: PLoS One. 2022 Nov 17;17(11):e0277680. doi: 10.1371/journal.pone.0277680 (PMC9671364; doi:10.1371/journal.pone.0277680)
Supplement: S1 File — (DOCX) [file pone.0277680.s001.docx]

**S1 File. Supplementary materials for Novel genotyping algorithms for rare variants significantly improve the accuracy of Applied Biosystems™ Axiom™ array genotyping calls: retrospective evaluation of UK Biobank array data**

| 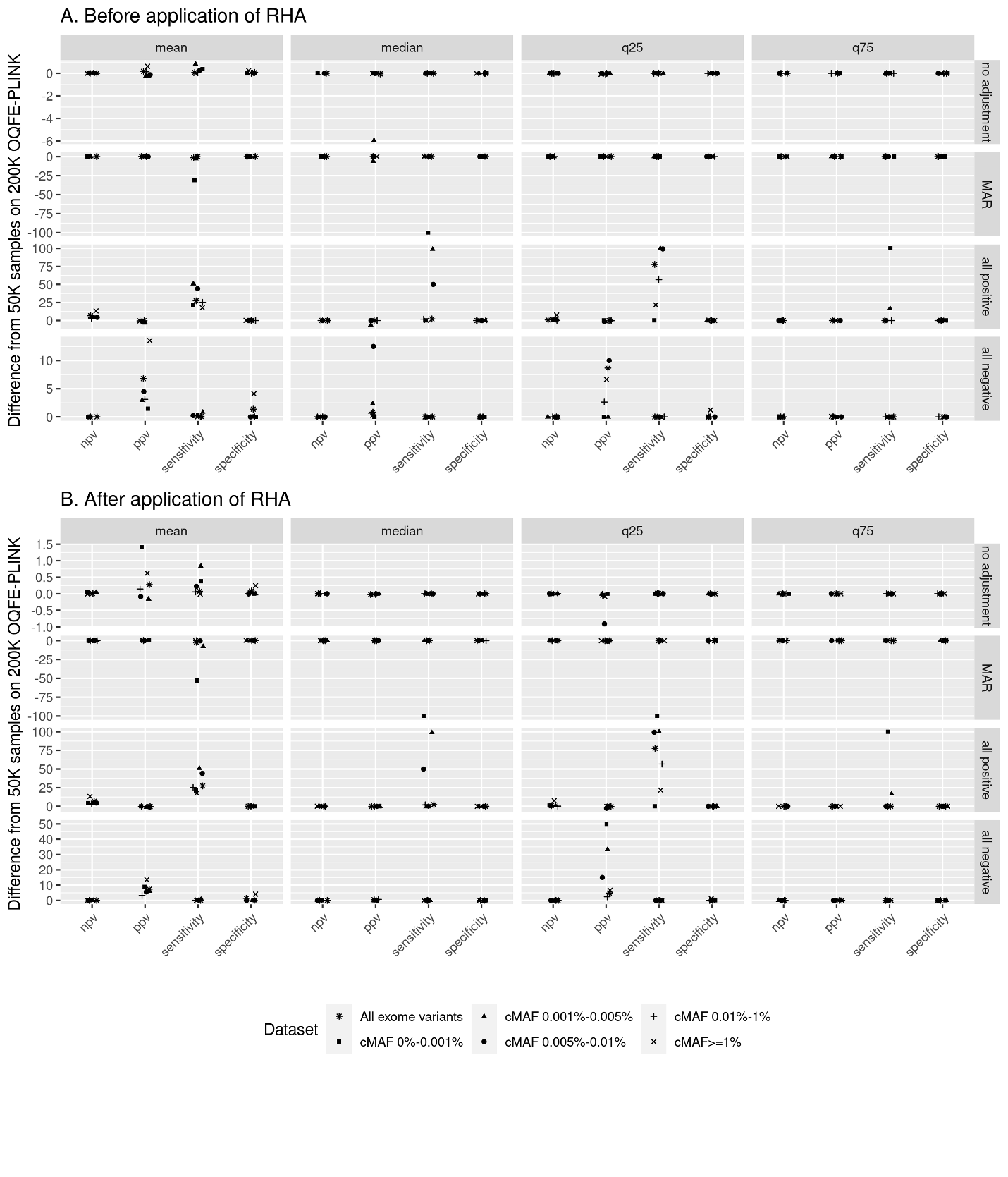 |
| --- |

**S1 Fig.** **Comparison of UK Biobank Axiom performance as assessed using the 50k FE-VCF dataset as reference to the performance as assessed using the portion of the 200k OQFE-PLINK dataset pertaining to the same samples.** The 50k FE-VCF has a significant portion of genotypes missing (Table S1). The 200k FE-PLINK dataset doesn’t suffer from this problem. To assess the bias in the complete-pair analysis that uses the 50k FE-VCF dataset as reference, we subtract the mean, median, first quartile (q25) and third quartile (q75) of the four performance metrics (npv=negative predictive value, ppv=positive predictive value, sensitivity and specificity) from the corresponding values as assessed using the 200k OQFE-PLINK dataset, restricted to the same 50k samples. Thus, a negative value means that we have over-estimated the true performance value. Differences were calculated for four possible adjustments that account for missing reference values: ‘no adjustment’ (complete-pair analysis); ‘MAR’= missing at random assumption,[1] i.e. positive predictive and negative predictive values are assumed correct while sensitivity and specificity are recalculated to account for the proportions of index test positives and negatives when including cases where reference is missing; ‘all positive’=all cases with missing reference are assumed positive; ‘all negative’=all cases with missing reference are assumed negative. Results are presented for all exome variants, as well as for each of the cMAF ranges both before (A) and after (B) applying the RHA algorithm. Assessment was restricted to variants present in both datasets. Please note differences in scale on the y-axis between the different rows.

| 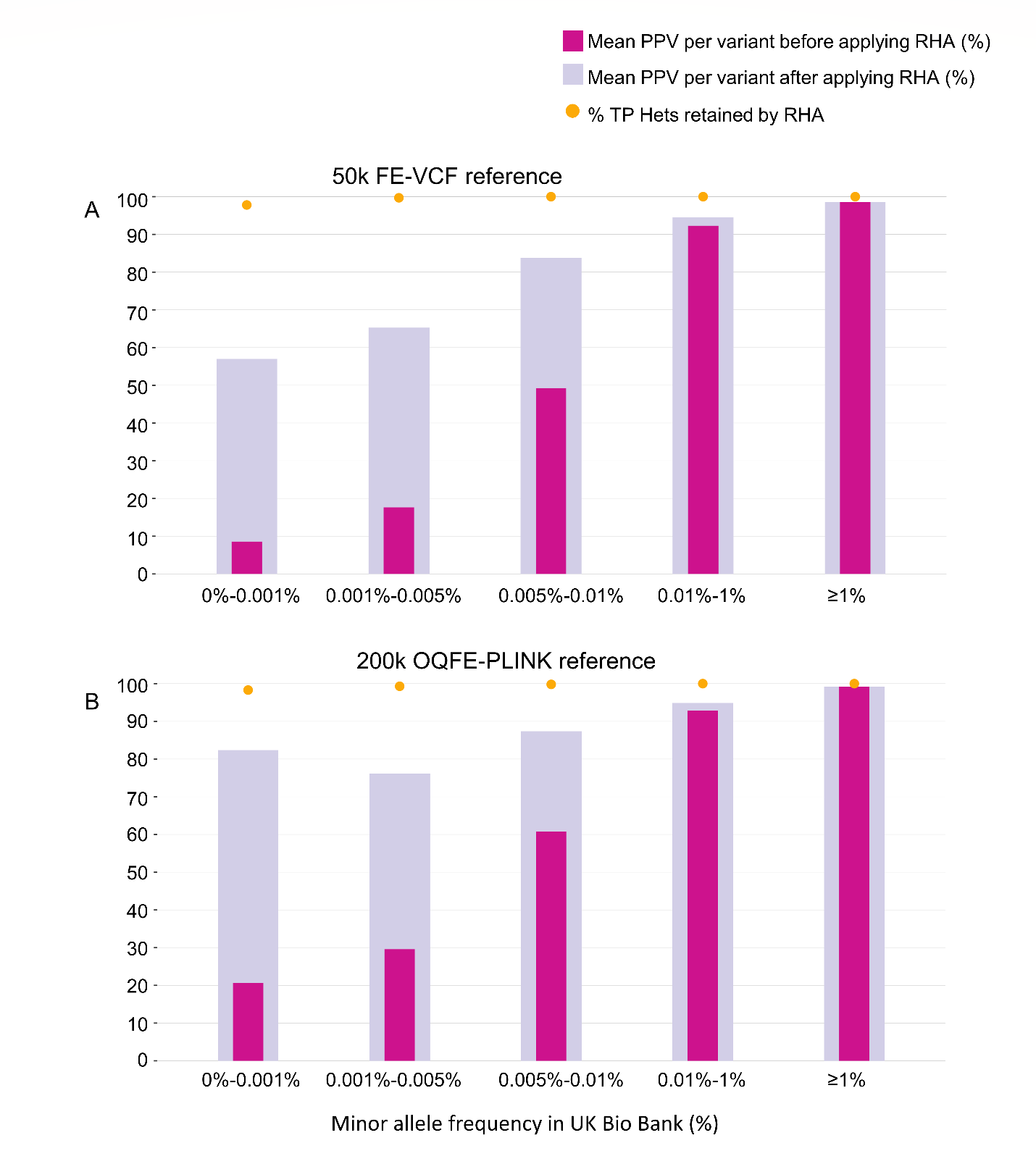 |
| --- |

**S2 Fig.** **Improvement in positive predictive value of rare variants on the UK BiLEVE Axiom array after application of RHA in the 50k and 200k data.** Bars indicate mean positive predictive value (PPV) of variants genotyped by UK Biobank Axiom array. The minor allele frequency ranges were calculated from the genotyping results (cMAF) before applying RHA. We used genotypes from exome datasets 50k FE-VCF (A) or 200k OQFE-PLINK (B) as truth, comparing performance before and after applying RHA. We also indicate the percentage of true positive heterozygous calls (TP hets) retained after applying RHA. Data for the UK BiLEVE Axiom array shows similar trends (S2 Fig). Note that for a given cMAF range, the number of variants contributing to the mean may be lower after the application of RHA because for some variants RHA eliminates all heterozygous predictions by the array, so that the positive predictive value cannot be calculated.

**Supplementary materials references**

1. Kosinski AS, Barnhart HX. A global sensitivity analysis of performance of a medical diagnostic test when verification bias is present. Stat Med. 2003;22: 2711–2721. doi:10.1002/sim.1517
