# Supplementary material for: Novel genotyping algorithms for rare variants significantly improve the accuracy of Applied Biosystems™ Axiom™ array genotyping calls: Retrospective evaluation of UK Biobank array data
Source: PLoS One. 2022 Nov 17;17(11):e0277680. doi: 10.1371/journal.pone.0277680 (PMC9671364; doi:10.1371/journal.pone.0277680)
Supplement: S5 Table — (DOCX) [file pone.0277680.s006.docx]

**S5 Table. Distributions of single nucleotide variants with coverage by both Axiom UK Biobank array and whole exome sequencing (WES) among the three Groups.**

| cMAF range | Total number of variants | Number of variants non-responsive in Axiom (Group 1) | Number of variants monomorphic in WES (Group 2) | Number of ‘other’ variants (Group 3) |
| --- | --- | --- | --- | --- |
| 0%-0.001% | 11,700 | 1,586 | 7,267 | 3,209 |
| 0.001%-0.005% | 14,619 | 541 | 5,082 | 9,093 |
| 0.005%-0.01% | 4,340 | 84 | 368 | 3,892 |
| 0.01%-1% | 40,350 | 4 | 303 | 40,044 |
| ≥1% | 28,957 | 0 | 29 | 28,928 |

Notes: for the first three rows the numbers in the three categories do not add up to the total. This is due to a relatively small number of variants (at most 5% of the variants in the cMAF range) that are both “non-responsive in Axiom” and “monomorphic in WES.” In Fig 5A these are counted in the ‘non-responsive in Axiom’. There are 427 variants that are polymorphic in the 50k EF-VCF data but are missing from the 200k OQFE-PLINK data; we do not consider these variants as ‘monomorphic in WES’
