# Supplementary material for: Novel genotyping algorithms for rare variants significantly improve the accuracy of Applied Biosystems™ Axiom™ array genotyping calls: Retrospective evaluation of UK Biobank array data
Source: PLoS One. 2022 Nov 17;17(11):e0277680. doi: 10.1371/journal.pone.0277680 (PMC9671364; doi:10.1371/journal.pone.0277680)
Supplement: S6 Table — (DOCX) [file pone.0277680.s007.docx]

**S6 Table. Effect of adding monoWES probesets on the positive predictive value of UK Biobank Axiom array**

| cMAF range | Before/  After application of RHA | Positive Predictive Value (%) | | | %TP Hets retained after RHA |
| --- | --- | --- | --- | --- | --- |
|  |  | Number of variants | Median (interquartile range) | Mean |  |
| All exome variants | Before | 96,620 | 99.0% (85.7%-99.9%) | 79.5% |  |
|  | After | 88,157 | 99.6% (97.4%-100.0%) | **90.3%** | >99.9% |
| cMAF 0%-0.001% | Before | 8,646 | 0.0% (0.0%- 0.0%) | 14.7% |  |
|  | After | 3,104 | 100.0% (0.0%-100.0%) | **57.1%** | 99.5% |
| cMAF  0.001%-0.005% | Before | 14,397 | 20.0% (0.0%- 80.0%) | 37.5% |  |
|  | After | 11,495 | 90.9% (0.0%-100.0%) | **61.7%** | 99.8% |
| cMAF  0.005%-0.01% | Before | 4,328 | 89.0% (61.1%- 97.2%) | 73.0% |  |
|  | After | 4,310 | 97.0% (80.0%-100.0%) | **80.9%** | 99.7% |
| cMAF  0.01%-1% | Before | 40,323 | 98.9% (96.4%- 99.8%) | 95.0% |  |
|  | After | 40,322 | 99.1% (97.0%- 99.8%) | **95.6%** | >99.9% |
| cMAF≥1% | Before | 28,926 | 99.9% (99.6%-100.0%) | 99.1% |  |
|  | After | 28,926 | 99.9% (99.6%-100.0%) | **99.1%** | 100.0% |

We used 200k OQFE-PLINK (n=195,447) as reference, adding we add variants monomorphic in 50k FE-VCF but absent from 200k OQFE-PLINK to the analysis of variants in 200k OQFE-PLINK. For these added variants, all positive predictions in the samples sequenced by whole exome sequencing are assumed to be false positives and they can therefore only contribute positive predictive values of 0 to the distribution (or be excluded from the distribution if they have no positive predictions). Results are split by the minor allele frequency calculated from the array before application of RHA (cMAF). We include the following summary statistics of the distribution of positive predictive values, calculated across all relevant single nucleotide variants: median, mean, and inter-quartile range. “Number of variants” is the number of variants the summary statistics have been calculated over. We also include “%TP hets retained after RHA”, which indicates the small drop in sensitivity imposed by the RHA algorithm.
