# Supplementary material for: Novel genotyping algorithms for rare variants significantly improve the accuracy of Applied Biosystems™ Axiom™ array genotyping calls: Retrospective evaluation of UK Biobank array data
Source: PLoS One. 2022 Nov 17;17(11):e0277680. doi: 10.1371/journal.pone.0277680 (PMC9671364; doi:10.1371/journal.pone.0277680)
Supplement: S8 Table — (DOCX) [file pone.0277680.s009.docx]

**S8 Table. Characterization of BRCA variant performance on the Axiom™ arrays used by UK Biobank.**

| Array type | UK BiLEVE | UK BiLEVE | UK Biobank |
| --- | --- | --- | --- |
| Cohort | 50k WES | 200k WES | 200k WES |
| Which BRCA variants? | Weedon et al.[1]  n = 1076 | Weedon et al.[1]  n = 99 | cMAF < 0.01%  n = 460 |
|  |  |  |  |
| TPs | 5 | 6 | 2528 |
| FNs | 0 | 1 | 454 |
| Exome Het  Array NoCall | 1 | 4 | 809 |
| Exome NoCall Array Het | 71 | 14 | 54 |
|  |  |  |  |
| Overall  Sensitivity | 100% | 85.7% | 84.8% |
| Variants with 100% sensitivity | 5 of 5 | 5 of 6 | 318 of 388 |
|  |  |  |  |
| Pre-RHA FPs | 739 | 132 | 2211 |
| Overall PPV | 0.67% | 4.3% | 53.4% |
|  |  |  |  |
| Post-RHA FPs | 343 | 67 | 598 |
| Overall PPV | 1.4% | 8.2% | 80.9% |
|  |  |  |  |
| TPs removed  by RHA | 0 | 0 | 3 (0.12%) |
| FPs removed  by RHA | 53.6% | 49.2% | 73.0% |

The variants studied by Weedon et al.[1] were graciously provided by the authors, and the data was re-analyzed to include only those variants that were present on the specified array. Each data point represents one heterozygous call in one sample. Some participants had more than one heterozygous call.
To provide additional data from the UK Biobank array, we also present results from all rare variants (cMAF < 0.01%) within the transcribed regions of *BRCA1* and *BRCA2.*

**References**

1. Weedon M, Jackson L, Harrison J, Ruth K, Tyrrell J, Hattersley A, et al. Use of SNP chips to detect rare pathogenic variants: retrospective, population based diagnostic evaluation. BMJ. 2021;372: n214. doi:10.1136/bmj.n214
